# The Association between Diet Quality and Health Status in Mobile Food Pantry Users in Northeastern Connecticut

**DOI:** 10.3390/nu14061302

**Published:** 2022-03-19

**Authors:** Dalia Marmash, Kyungho Ha, Junichi R. Sakaki, Isabella Gorski, Brazil Rule, Michael Puglisi, Ock K. Chun

**Affiliations:** 1 Department of Nutritional Sciences, University of Connecticut, Storrs, CT 06269, USA; dalia.marmash@uconn.edu (D.M.); junichi.sakaki@uconn.edu (J.R.S.); isabella.gorski@uconn.edu (I.G.); brazil.rule@uconn.edu (B.R.); michael.puglisi@uconn.edu (M.P.); 2 Department of Food Science and Nutrition, Jeju National University, Jeju 63243, Korea; kyungho.ha@jejunu.ac.kr

**Keywords:** mobile food pantry, diet quality, health status, low-income, food insecurity, chronic disease

## Abstract

Low-income Americans tend to have poor diet quality and disease prevalence overall. Mobile food pantries aim to improve these outcomes, and have rarely been studied. This cross-sectional study aimed to evaluate the association between diet quality and health status in mobile food pantry users. Data were collected from two mobile food pantry sites in Northeastern Connecticut (*n* = 83). Sociodemographic food security and diet quality data were collected. Overall, diet quality was low among all participants with intakes of fruits, vegetables, and whole grains of concern. Participant adherence to the 2020–2025 US Dietary Guidelines were low, with no participants meeting recommendations for whole grains. Obesity, diabetes, and hypertension prevalence in this population exceeded national averages. After adjusting for covariates, hypertension was associated with higher dairy and added sugar intake, as well as a greater intake of added sugar from sugar-sweetened beverages (*p* < 0.05). Although results were not statistically significant, participants with obesity, diabetes, and hypertension showed a trend of having lower adherence to the guidelines than those without these chronic diseases. Questions assessing participant interest in changing their diets were also posed, indicating overall high interest in learning about current diet quality and weight improvement.

## 1. Introduction

In 2019, 10.5% of Americans were considered to be food insecure—defined as not having enough, or the right types, of food to sustain an active and healthy lifestyle [1]. Although Connecticut is not among the states with the highest rates of poverty in the United States, it has more concentrated poverty and wealth than most states. According to the DataHaven Study in 2019 [2], this “wealth and income polarization” expanded at a rate three times faster than the national average in some regions of Connecticut. Furthermore, a 2014 survey of Connecticut food pantry users found that over 60% of individuals had to choose between buying food or paying for other essentials such as rent and medical care [3]. This has resulted in the reliance on less expensive, energy dense, and nutrient-poor foods with a decrease in food variety and quantity among this population [4,5,6,7].

There are several programs in place to assist food insecure and low-income individuals. Food pantries, part of the emergency food system, are designed to relieve short-term food insecurity. In much of the United States, however, food pantries have become a major, recurrent source of food for low-income individuals suffering from chronic food insecurity [8]. Food pantries rely primarily on volunteers and donations, leading to vast variations in food quality, storage capacity, and distribution technique [9]. Mobile food pantries, where refrigerated trucks bring perishable foods to communities on a scheduled basis, have become an increasingly popular method of food distribution. This allows for a more wide-spread supply of perishable food such as dairy, produce, and proteins [3], serving many of the highest-need households facing a number of unique medical and mobility challenges—including seniors. Thus, mobile pantries have a great potential to function as a place to access food for those in need and to provide them with health promotion interventions. However, users of mobile food pantries have rarely been studied, and assumptions about their diet quality and health are often drawn from information gathered on traditional food pantries and their participants. Due to the differences in available food and the increased accessibility to those with mobility and transportation concerns, an assessment of this population is required.

In general, traditional food pantry users have lower quality diets than the average American—with fruits, vegetables, whole grains, dairy, and related micronutrients of particular concern [10,11,12,13,14]. This low-quality dietary pattern is associated with an increased risk of a variety of chronic diseases, namely obesity, hypertension, and diabetes [15]. Among food-insecure populations, the prevalence of these diseases exceeds national averages [16,17]. A study among 711 patients with diabetes found that those classified as food insecure were more likely to have poor glycemic control, less diabetes related self-efficacy, and more trouble affording a diabetic-friendly diet [18]. Food insecurity has also been associated with a 20% higher rate of hypertension compared to food secure individuals [19]. Although a direct association between food insecurity and overweight/obesity has not been conclusively established, the consumption of a low-quality diet is undoubtedly linked with excess adiposity.

While there are various studies evaluating the challenges food pantry clients face, there is a lack of literature focusing on mobile food pantry users. The differences in distribution method, food offered, and populations served make an assessment of mobile food pantry users essential. Thus, the current study aimed to characterize mobile food pantry users in Northeastern Connecticut, and to specifically evaluate their diet quality, disease prevalence, and health education interest.

## 2. Materials and Methods

### 2.1. Study Design and Participants

Participants were recruited from two mobile food pantry sites in Willimantic, Windham County, Connecticut, which were each visited once. Windham County was selected due to its lowest median income in Connecticut [20] as well as highest prevalence of diabetes (11%) and adult obesity (31%) [21]. There is a poverty rate of 27.8% and a food insecurity rate of 16.4%—higher than national and state averages [22]. Participants were recruited while waiting in line at the mobile food pantry, and were invited to participate if they were at least 19 years of age and had visited a food pantry at least once prior to recruitment (to distinguish regular food pantry users from those who have not utilized a food pantry previously). Consent forms were explained by research staff and signed by participants prior to study commencement. The research protocol was approved by the BLINDED Institutional Review Board as protocol #H19-206.

Three questionnaires were administered orally by research staff prior to the distribution of food at the mobile pantry. The first survey collected sociodemographic and self-reported health data. Then, the US Department of Agriculture’s (USDA) Household Food Security Questionnaire and the National Cancer Institute’s (NCI) Dietary Screener Questionnaire (DSQ) were administered. Participants were given a USD 20 Walmart gift-card for the completion of the first day of interviews. In total, 83 participants completed the first day of interviews.

### 2.2. Sociodemographic Characteristics

Sociodemographic characteristics were collected via a study-specific questionnaire. Poverty class was determined using median household incomes compared to published poverty threshold values considering family size, the number of older adults, and the number of children under 18 [23]. Participants were additionally sorted into poverty classes based on published poverty thresholds: above poverty (greater than thresholds), poverty (less than or equal to thresholds, but greater than 50% of thresholds), and extreme poverty (less than 50% of thresholds). Food security categorizations were based on participant scores on the 18 question USDA Household Food Security survey. According to USDA guidelines, a score of 2–4 corresponds to low food security and a score of 5 or above to very low food security [24]. Due to the small sample size, those falling into the marginal and high food security categorizations were combined and labeled “food secure” during analysis.

### 2.3. Health Status Assessment

The participants’ height, weight, and waist circumference were measured in a private area out of view of others. Jackets and shoes were removed, and weight was measured using a digital scale. Waist circumference was measured using a measuring tape directly above the ileac crest, with bulky clothing removed. Height was measured without shoes using a stadiometer. Diabetes and hypertension status was self-reported, and defined as having been diagnosed by a physician. Weight status and obesity class were determined using anthropometric measures taken on the first day of interviews, as described previously, and BMI cut-off values (normal weight: 18.5 < BMI < 24.9; overweight: 25 < BMI < 29.9; obese class 1: 30 ≤ BMI < 35; obese class 2: 35 ≤ BMI < 40; obese class 3: BMI > 40). For comparison of participant weight and health status, data from the Center for Disease Control from 2013–2016 was used, which was collected using the National Health and Nutrition Examination Survey [25,26,27].

### 2.4. Dietary Assessment

To assess recent diet quality in the 83 participants, the 26-item DSQ was used to estimate daily intake of various foods over the previous 30 days. It allows for general dietary trends to be observed, although it may not necessarily be indicative of long-term intake, especially in food insecure individuals. The predicted intakes of fruits and vegetables, dairy, added sugars, and whole grains were estimated according to scoring algorithms developed by the NCI [26]. Predicted intakes of fruits and vegetables were compared to the US Dietary Guidelines for Americans (USDGA) (2 cups/day for fruits and 2.5 cups/day for vegetables) [28], and the percentage of participants meeting the guidelines was calculated.

### 2.5. Interest in Learning about Nutrition and Improving Weight Status

Participants were asked four questions to assess interest in learning about health behavior changes. Participants rated interest in knowing more about current diet quality, knowing the types of foods to consume more or less of, learning some healthy cooking recipes, and learning how to improve weight status. Answers included “very uninterested”, “somewhat uninterested”, “neutral”, “somewhat interested”, and “very interested”. Answers were then converted into numerical values from 1–5, with 5 translating to “very interested”.

### 2.6. Data Analysis

Values are presented as mean ± standard deviation or as *n* (%). To compare differences in intakes of food groups by presence of chronic disease including obesity, diabetes, and hypertension, we utilized a general linear model, adjusting for age, sex, and race. To compare differences in percentage of participants adhering to USDGA 2020–2025 for fruits and vegetable intake by disease status, we conducted Fisher’s exact test, but there was no significant difference in adherence. To compare differences in participant interest in health education by disease status, we used the Wilcoxon rank-sum test and Kruskal–Wallis test due to the small sample size. All *p* values were two-sided and *p* < 0.05 was considered as statistically significant. Data analysis was conducted using SAS software (version 9.4; SAS Institute, Cary, NC, USA).

## 3. Results

### 3.1. Sociodemographic Characteristics

The mobile food pantry users (*n* = 83) sampled from Windham County, Connecticut were predominantly female, Latinx, and over the age of 44 (Table 1). Participants were also largely unemployed or not seeking employment, with 90.2% of participants making below USD 30,000 annually. Almost two-thirds of the participants were considered to be in poverty or extreme poverty, with a similar proportion of participants falling into the low or very low food secure categories. The majority of participants (68%) had low or very low food security.

### 3.2. Health Status of Participants versus National Averages

Participant hypertension, diabetes, and obesity prevalence were higher than national averages among the general adult public (Table 2). Prevalence of diabetes was almost three times the national average, and more than ninety percent of the study participants were overweight or obese. The data were also compared with national data of those below the federal poverty line—aligning more closely to the socioeconomic status of the study participants. When compared to the data of lower income Americans, study participants still had higher prevalence of obesity, diabetes, and hypertension.

### 3.3. Diet Quality and Disease Prevalence

**Figure 1:**
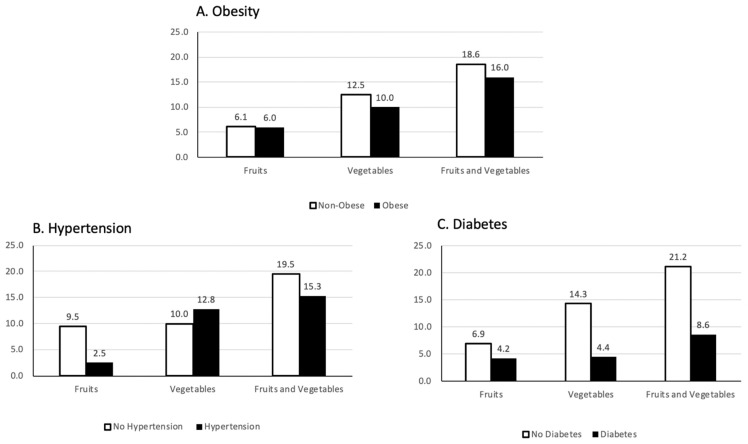
Percentage of participants adhering to the USDGA 2020–2025 recommendations of daily fruit (2 cups) and vegetable (2.5 cups) consumption by self-reported chronic disease among food pantry study participants (*n* = 83). (**A**) Obesity; (**B**) hypertension; (**C**) diabetes.

Diet quality was assessed using DSQ data from the participants and evaluated by chronic disease prevalence. Overall, compared to the Dietary Guidelines for American 2020–2025, fruit, vegetable, and whole grain intake was extremely low among participants (Table 3). Furthermore, added sugar intake exceeded recommendations in this sample (Table 3). After adjusting for age, sex, and race, hypertension was associated with higher dairy and added sugar intake, as well as a greater intake of added sugar from sugar-sweetened beverages (*p* < 0.05) (Table 3). Figure 1 shows adherence to the DGA 2020–2025 for fruits, vegetables, and fruits and vegetables by health status. Overall, as reported in Table 3, adherence to the DGA was low. Although they were not statistically significant, largely those with obesity, diabetes, and hypertension showed a trend of having lower adherence to the guidelines than those without these chronic diseases. Specifically, no participants with class 3 obesity (BMI > 40) had intake adhering to the DGA (data not shown). Furthermore, no participants, regardless of disease status, had adequate intakes of whole grains (data not shown).

### 3.4. Participant Interest in Learning about Health Behavior Changes

**Figure 2:**
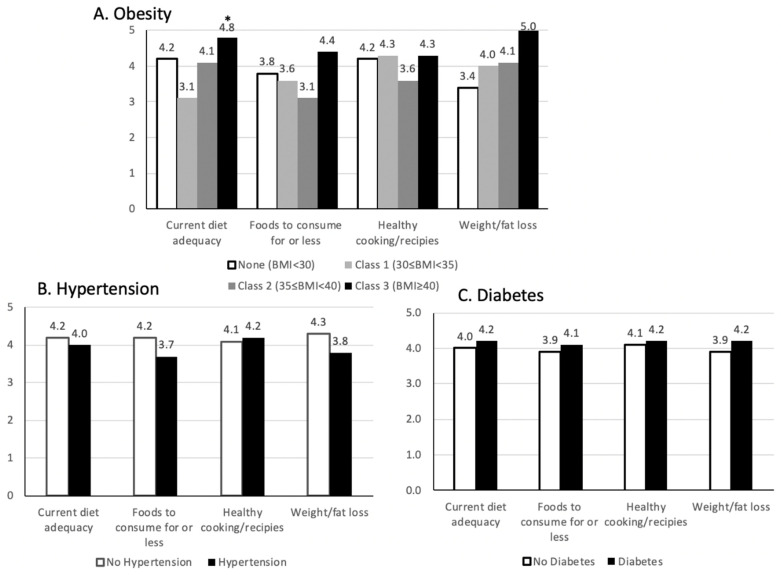
Participant (*n* = 83) interest in learning about healthy eating topics. Participant disease status: (**A**) Obesity, (**B**) hypertension, (**C**) diabetes. *Y*-axis scale indicates participant interest in learning about the topics listed on the *x*-axis. 0 translates to “not-interested”, and 5 translates to “very interested”. * Indicates statistical significance using Wilcoxon rank-sum and Kruskal–Wallis tests (*p* < 0.05).

Overall, there was significant interest in learning about current diet adequacy, healthy cooking, weight loss, and diet improvement, shown in Figure 2. All participants with class 3 obesity were extremely interested in learning about weight loss. Those with class 3 obesity were also significantly more interested in learning about current diet adequacy.

## 4. Discussion

This cross-sectional study of mobile food pantry users in Northeastern Connecticut aimed to assess the associations between chronic disease prevalence and diet quality, as well as participant interest concerning diet and health improvement. Within this sample, obesity, hypertension, and diabetes prevalence exceeded national averages. Diet quality was low among all participants with intakes of fruits, vegetables, whole grains, and dairy of particular concern. Hypertension was associated with increased dairy, total added sugar, and added sugar from sugar-sweetened beverages intake in our sample.

Poor diet quality has been proven to impact chronic disease prevalence, even if calorie intake is not significantly increased [29]. In Canadian adults, higher diet quality assessed by Healthy Eating Index scores translated to lower self-reported chronic disease incidence in both men and women [29]. Chronic disease has also been associated with food insecurity, with low-income individuals being more likely to have risk factors for chronic disease, such as hypercholesteremia and hypertension [19,30]. Furthermore, numerous studies have observed inadequate intake of nutritious foods among food insecure populations—including fresh produce and dairy [10,11,12,13]. These observations could be due to the high cost of these food groups, as well as lack of access in lower income areas [31].

The current study similarly observed overall low intake of fruits, vegetables, whole grains, and dairy among participants. Consumption of components of moderation, such as added sugars and sodium exceeded recommendations in this sample. As mentioned in previous studies, low quality, calorically dense foods are often less expensive and more accessible to low-income populations, explaining the elevated intake observed [6,7,31]. These disparities in diet quality could be a contributing factor to the development of disease among these participants. For example, in the analysis of DSQ data, higher intakes of added sugar and added sugar from sugar-sweetened beverages were associated with hypertension. Diets high in added sugars increase one’s risk of developing hypertension, in combination with low fruit, vegetable, and whole grain intake [32,33,34].

Participant interest in learning more about nutrition and health was high. All participants with class 3 obesity were interested in learning about weight loss. Class 3 obesity was also associated with greater interest in learning about current diet adequacy. This information is useful in determining the stage of change, as outlined in the transtheoretical model, that participants are currently experiencing [35]. It is important for participants to be willing to change their behaviors, or to at least contemplate change, for successful interventions to take place. Interventions focused on educating participants about proper nutrition and cooking techniques seem to be the most successful in food insecure populations [36,37,38]. There have also been interventions focused on simply rearranging foods in food pantries—making healthier choices more visible and appealing—which seem to be effective in improving diet quality [39]. Interventions should take into account the various barriers faced by food insecure populations to effectively change their dietary behaviors. Topics such as shopping on a budget and preparing recipes that are inexpensive, but healthy and simple to prepare would be of particular benefit.

The current study is one of the few assessing relationships between dietary and disease status in mobile food pantry users. The unique features of mobile food pantries—fresher foods, greater accessibility—make extrapolation of previous studies in traditional food pantry users difficult. Important conclusions, such as a strikingly high prevalence of chronic disease, low diet quality, and high interest in learning how to improve diet and health status, will help researchers better understand this population’s specific needs.

There are several strengths of the current study. First, all surveys were administered by research personnel, ensuring participant understanding of all questions. Furthermore, the use of the DSQ allowed for the estimation of participants’ usual dietary intake over the last 30 days. However, the small sample size is a limitation, leading to less generalizable but more conservative findings. Furthermore, the self-reporting of disease status is limited by healthcare access, which may be limited in this low-income population, which may result in underreporting due to misclassification bias. Another limitation of the current study is the lack of diversity of our sample, with the majority of participants being older, female, and/or Hispanic/Latinx. Furthermore, due to the nature of the dietary assessment methods, recall bias may be a source of error. The current data still provides important insights into disease and diet quality in this population.

This novel assessment has elucidated the specific nutrition and health concerns of mobile food pantry users of Northeastern Connecticut. Future nutrition intervention strategies in this population should consider the high chronic disease prevalence and overall low diet quality of participants. Interventions should focus on cost effective diet improvement strategies, such as promoting frozen or canned fruits and vegetables. An emphasis should also be placed on healthy weight loss and reducing sodium, added sugar, and saturated fat intake, while increasing the consumption of fiber rich foods. Mobile food pantry users in Northeastern Connecticut have generally low diet quality and higher than average chronic disease prevalence, with relationships present between dietary intake and disease status. These findings indicate that health behavioral interventions tailored for individuals’ nutrition and health status are much needed for and sought by this highly vulnerable population.

## 5. Conclusions

This assessment of the diet quality and health status of mobile food pantry users will aide future efforts to develop an effective intervention in this population. Their unique sociodemographic make-up, overall poor diet quality, and their high prevalence of obesity, diabetes, and hypertension are important factors to consider for future studies. Tailoring nutrition messaging to those with these chronic diseases would be useful for this population. It is also important to consider the largely Hispanic make-up of this population. Using culturally appropriate materials will further ensure the success of a future intervention. Their high interest in learning more about healthy eating is also useful, suggesting future interventions would be highly accepted in this population. It is important to understand the needs of the target population prior to the development and implementation of an intervention.

## Figures and Tables

**Table 1:** Sociodemographic characteristics of study participants (*n* = 83) recruited from mobile food pantries in Windham County, Connecticut.

Characteristics	*n*	%
Sex		
Male	16	19.3
Female	67	80.7
Age (years)		
19–30	8	9.6
31–44	13	15.7
45–64	41	49.4
≥65	21	25.3
Race		
Hispanic or Latinx	53	63.9
Non-Hispanic White	24	28.9
Other ^1^	6	7.2
Education		
Less than high school	32	38.6
Completed high school	29	34.9
Some college or more	22	26.5
Employment		
Employed	18	21.7
Unemployed	28	33.7
Not seeking employment ^2^	37	44.6
Annual Household Income ^3^		
Less than USD 5000	11	13.4
USD 5001–USD 10,000	24	29.3
USD 10,001–USD 15,000	19	23.2
USD 15,001–USD 30,000	20	24.4
More than USD 30,000	8	9.8
Poverty status ^3,4^		
Above poverty	29	35.4
Poverty	32	39.0
Extreme poverty	21	25.6
Food security status ^5^		
Food security	25	30.1
Low food security	28	33.7
Very low food security	30	36.1

^1^ Non-Hispanic Black or African American, Asian or Asian American, or Multi-Racial. ^2^ Retired, full-time homemaker, or on disability. ^3^ One participant did not report household income. ^4^ PIR (poverty income ratio) above published thresholds (considering family size, the number of older adults, and the number of children under 18) or below the threshold [21]. ^5^ Food security was defined according to the USDA Household Food Security Questionnaire [22].

**Table 2:** Comparison of health status including obesity, diabetes, and hypertension between current food pantry study participants and average US adults or other food pantry participants (%).

Characteristics	Food Pantry Study Participants (*n* = 83)	Adult National Average ^1^	Adults in 100–199% of Poverty Line ^1–3^
Body weight			
Underweight (BMI < 18.5)	2.4	1.7	-
Normal (18.5 ≤ BMI < 25)	3.6	27.7	25.1
Overweight (25 ≤ BMI < 30)	33.7	31.8	-
Obese (BMI ≥ 30)	60.2	38.8	41.6
Class 1 (30 ≤ BMI < 35)	44.0	21.2	22.0
Class 2 (35 ≤ BMI < 40)	26.0	9.9	9.9
Class 3 (BMI ≥ 40)	30.0	7.7	9.8
Diabetes			
Yes	29.3	10.9	14.6
No	70.7	89.1	85.4
Hypertension			
Yes	48.8	32.2	36.6
No	51.2	67.8	63.4

^1^ CDC National Health Data 2015–2016 [23]. ^2^ CDC National Health Data Diabetes 2013–2016 [25]. ^3^ CDC National Health Data Hypertension 2013–2016 [24].

**Table 3:** Predicted intakes (mean ± SD) of food groups by self-reported chronic disease among food pantry study participants (*n* = 83).

		Obesity Class ^1^		Diabetes ^2^		Hypertension ^2^	
	Recommended Intake ^3^	Non-Obese (*n* = 33)	Obese (*n* = 50)	No (*n* = 58)	Yes (*n* = 24)	No (*n* = 42)	Yes (*n* = 40)
Whole grain (oz)	6.0	0.9 ± 0.1	0.8 ± 0.1	0.8 ± 0.1	1.0 ± 0.1	0.8 ± 0.1	0.9 ± 0.1
Dairy (cup)	3.0	2.0 ± 0.2	1.6 ± 0.2	1.8 ± 0.2	2.0 ± 0.2	1.6 ± 0.2 *	2.0 ± 0.2
Fruits and vegetables including legumes and French fries (cup)	4.5	3.0 ± 0.2	3.0 ± 0.2	3.1 ± 0.2	2.9 ± 0.2	3.0 ± 0.2	3.1 ± 0.2
Vegetables including legumes and including French fries (cup)	4.5	1.9 ± 0.1	1.9 ± 0.1	2.0 ± 0.1	1.8 ± 0.1	1.9 ± 0.1	1.9 ± 0.1
Vegetables including legumes and excluding fries (cup)	2.5	1.8 ± 0.1	1.8 ± 0.2	1.9 ± 0.1	1.7 ± 0.2	1.8 ± 0.1	1.8 ± 0.1
Fruits (cup)	2.0	1.1 ± 0.1	1.0 ± 0.1	1.1 ± 0.1	1.1 ± 0.2	1.0 ± 0.1	1.2 ± 0.1
Added sugar (tsp)	<12.0	19.6 ± 1.7	15.9 ± 1.8	18.1 ± 1.6	17.9 ± 2.2	15.7 ± 1.8 *	20.0 ± 1.7
Added sugar from sugar-sweetened beverages (tsp)	<12.0	8.9 ± 1.4	6.9 ± 1.5	8.0 ± 1.3	8.8 ± 1.8	5.7 ± 1.4 *	10.3 ± 1.4

All values are adjusted mean ± standard error after adjusting for age, sex, and race. * Indicates statistical significance of differences in food group intakes using general linear model (*p* < 0.05). ^1^ Non-obese: BMI < 30; obese: ≥30). ^2^ One participant did not report prevalence of diabetes or hypertension. ^3^ Based on the United States Dietary Guidelines for Americans 2020–2025. Minimum recommended intakes listed for whole grains, dairy, fruits and vegetables and maximum recommended intakes listed for added sugar and added sugar from sugar-sweetened beverages.

## Data Availability

Data is contained within the article.
